# Retraction: Identification of a Novel Jasmonate-Responsive Element in the *AtJMT* Promoter and Its Binding Protein for *AtJMT* Repression

**DOI:** 10.1371/journal.pone.0242041

**Published:** 2020-11-03

**Authors:** The PLOS ONE Editors

Following the publication of this article [1], concerns were raised regarding the data presented in multiple figures. Specifically,

- The bands presented for the Total RNA results in Fig 1A and 1B appear similar. Although some minor differences can be detected at pixel level, the corresponding author was unable to provide the original uncropped data underlying to support the results presented in the published figure.
- Similarities have been observed between blot data presented in multiple figures, including:
  ○ Fig 2A: the MeJA panels JP4.5, JP4.0, JP3.5, JP3.0 and JP 2.5.
  ○ Fig 2A and 2B: the MeJA panels JP1.5 and JP2200.
  ○ Fig 2B: when rotated 180° the MeJA panel JP2280 appears similar to JP2100.
  ○ Fig 3A: when rotated 180° the MeJA panel NP3.0 appears similar to NP2.0.
  ○ Fig 9A: the AtBBD1 panels *atbbd1* (0-6h) and *atbbd1 atbbd2* (0-6h).
  ○ Fig 9A: the Total RNA panels Col-0 (0-6h), *atbbd1* (0-6h), and *atbbd1 atbbd2* (0-6h).
  ○ Fig 9A and 9B: the 0-3h lanes of the total RNA panels representing Col-0, *atbbd1*, and *atbbd1 atbbd2* (0-6h) and the Total RNA (Col-0, OX4, and OX13) panel.
  ○ Fig 9C: the lanes OX4 (0h), OX4 (6h), and OX13 (6h) in the *AtBBD1* panel.
  ○ Fig 9C: the lanes OX4 (1h), OX13 (0h), and OX13 (1h) in the *AtBBD1* panel.
  ○ Fig 9C: the lanes OX4 (3h) and OX13 (3h) in the *AtBBD1* panel.
  ○ Fig 9C: the lanes Col-0 (3h), OX4 (1h), and OX13 (1h) in the *JMT* panel.
  ○ Fig 9C: the lanes Col-0 (6h) and OX4 (0h) in the *JMT* panel.
  ○ Fig 9C: the Total RNA OX4 (0-6h) and OX13 (0-6h) panels
  ○ S4 Fig: In the *JMT* panel the *jin 1–8* (0h) lane looks similar to the *jin 1–7* (6h) lane when flipped.
  ○ S4 Fig: the lanes *jin 1–7* (0h) and the *jin 1–8* (6h) in the *JMT* panel.
  ○ S4 Fig: the first half of the *TUB2* panel (lanes Col-0 (0-6h) and lanes *jin 1–7* (0h, 1h)) and the second half of the *TUB2* panel (lanes *jin 1–7* (3h, 6h) and lanes *jin 1–8* (0-6h)).
  ○ S5 Fig: the lanes *AtBBD1* (0h), *AtBBD1* (3-12h), *AtBBD2* (0h), and *AtBBD2* (3h and 6h) in the MeJA panel appear similar to the lanes *AtBBD1* (0h), *AtBBD1* (3-12h), *AtBBD2* (0h), and *AtBBD2* (3h and 6h) in the ABA panel, respectively.
  ○ S5 Fig: the lane *AtBBD2* (12h) in the MeJA panel and the lanes *AtBBD2* (1h and 12h) in the ABA panel.
  ○ S6 Fig: the lanes *atbbd2* (0h) and *atbbd2* (9h) in the *AtBBD2* panel.
  ○ S6 Fig: the lanes Col-0 (1h and 3h) and the lanes *atbbd2* (1h and 3h) in the *JMT* panel, respectively.
  ○ S6 Fig: the lanes Col-0 (9h), *atbbd2* (0h), and *atbbd2* (9h) in the *JMT* panel.
- When adjusting the colour levels discrepancies have been observed in the following panels:
  ○ Fig 1B: Horizontal and vertical discrepancies around the first band of the *GUS* panel.
  ○ Fig 9A: vertical discrepancies around the 6h lane of the *JMT* Col-0 panel.
  ○ S4 Fig: In the *JMT* panel, the background of the Col-0 (6h) lane appears similar to the background of the *jin 1–8* (0h) lane, and background of the *jin 1–7* (6h) lane when flipped.
- Similarities have been observed between colony data presented in multiple figures, including:
  ○ Fig 5A and 5B: the Fig 5A Control panel, the c panel and the a7 panel.
  ○ Fig 5A and 5B: the abc panel and the a8 panel. In addition, these panels appear similar to the a panel and the a5 panel when rotated 180°.
  ○ Fig 5A and 5B: the b panel and the a6 panel.
  ○ Fig 5C: the Control, M1, and M3 panels.
  ○ Fig 5D: the Control panel and CM3 panel.
  ○ Fig 5D: the CM5, CM7 and CMR panels.
  ○ Fig 5D/Fig 6: the JARE panel and the AtBBD2 P_NTR1_ panel when flipped.
  ○ Fig 6/S7 Fig: the AtBBD1 P_NTR1_ panel and the AtBBD2 AtJAZ1 panel.
  ○ Fig 6/S7 Fig: the AtBBD1 P_JMT_ panel and the AtBBD2 AtJAZ4 panel.
  ○ Fig 8A: the JAZ3, JAZ6, JAZ8, JAZ9, JAZ10, JAZ11 and JAZ12 panels.

The uncropped underlying data for the published figures are not available and the concerns have not been clarified. The authors provided some additional data, but they did not resolve the above concerns and in some cases the files received post-publication raised additional questions about data reporting and management. In light of the concerns affecting multiple figure panels that question the integrity of these data, the *PLOS ONE* Editors retract this article.

SYY agreed with the retraction. YJK and CJ either could not be reached or did not respond. JSS, YC, JSL, JKK, JTS, and YDC did not agree with the retraction.
